# Thermochemically activated carbon as an electrode material for supercapacitors

**DOI:** 10.1186/s11671-015-0762-1

**Published:** 2015-02-12

**Authors:** Bogdan K Ostafiychuk, Ivan M Budzulyak, Bogdan I Rachiy, Vitalii M Vashchynsky, Volodymyr I Mandzyuk, Roman P Lisovsky, Lyudmyla O Shyyko

**Affiliations:** Vasyl Stefanyk PreCarpathian National University, 57 Shevchenko Str., Ivano-Frankivsk, 76018 Ukraine

**Keywords:** Nanoporous carbon material, Potassium hydroxide, Chemical activation, Double electric layer, Electrochemical capacitor, 81.05.Uw, 88.80.Fh, 82.47.Uv

## Abstract

The results of electrochemical studies of nanoporous carbon as electrode material for electrochemical capacitors (EC) are presented in this work. Nanoporous carbon material (NCM) was obtained from the raw materials of plant origin by carbonization and subsequent activation in potassium hydroxide. It is established that there is an optimal ratio of 1:1 between content of KOH and carbon material at chemical activation, while the maximum specific capacity of NCM is 180 F/g. An equivalent electrical circuit, which allows modeling of the impedance spectra in the frequency range of 10^−2^ to 10^5^ Hz, is proposed, and a physical interpretation of each element of the electrical circuit is presented.

## Background

One of the modern trends in the development of rechargeable energy sources is the creation of highly efficient electrochemical capacitors (EC), working on the charge/discharge principle of the electrical double layer (EDL) in polarized electrodes with large specific surface area. The EDL charge/discharge mechanism is reversible and reproducible up to thousands of cycles, each of which lasts a fraction of a second [1,2]. Great power density, long life, environmentally friendly manufacturing techniques and utilization, easy adaptation, and use in technology are the reasons of their expanding scope. In particular, electric cars and hybrid cars use EC as a supplement to the existing battery due to their ability to take on large peak loads. In addition, they are used to start diesel engines and internal combustion engines, in the energy recovery systems of the electric rise in municipal electric transport, etc. EC is a strategic product for improving the efficient use of electricity [3].

The value of the specific capacitance of a capacitor depends on the type of the electrolyte, the structure and condition of the developed surface of the electrode material, and its pore size distribution. The electrolyte defines the internal resistance (IR) of EC and the operating voltage that should not exceed the expansion potential of the solvent [4].

The unique ability of carbon atoms to form the valence states of different hybridizations of atomic orbitals creates prerequisites for studying of the technological conditions of their receipt through carbonization of the starting materials and the following chemical activation. As a result of the carbonization of raw materials from plants, we obtained material containing 90% of carbon. In this case, the carbon atoms form mainly sp^2^π hybridization (although, in general, it needs to say the mixed hybridization) with a complex framework of condensed aromatic layers. The degree of graphitization and amorphousness of the obtained carbon structure depends on the temperature and chemical treatment conditions. The surface of the obtained nanoporous carbon material (NCM) is significantly different according to the method of chemical activation. The final properties of the obtained NCM depend on several factors such as temperature of carbonization of the feedstock and the environment in which it occurs, future conditions, and modes of the chemical activation. Electrodes based on NCM are separated by a separator and placed in the electrolyte. They are an essential part of EC and working on the EDL charge/discharge principle at the interface of electrode ║ electrolyte. Electrochemical charging process can be represented as ${E}_S+{E}_S+{C}^{+}{A}^{-}\underset{Disch \arg e}{\overset{Ch \arg e}{\rightleftharpoons }}{E}_S\parallel {A}^{-}+{E}_S\parallel {C}^{+}$*,* where *Е*S is the surface of NCM porous structure, *C*^*+*^ and *А*^*−*^ are cations and anions of the electrolyte, and ║ is EDL which accumulates charge through the physical adsorption mechanism [5].

The aim of this work was to determine the effect of chemical activation of carbon carbonized with potassium hydroxide on capacitive characteristics of capacitors formed on the obtained material.

## Methods

As the active material, we used NCM derived from raw materials of plants by carbonization and activation with potassium hydroxide. Dried apricot seeds as a feedstock were crushed to fractions of 0.25 to 1 mm and carbonized in a close furnace at 250°C to 350°C with a heating rate of 10°C/min. The resulting carbon was mechanically crushed to fractions of 200 to 250 μm and mixed with potassium hydroxide and water in a weight ratio: *X*_*К*_ = 1, 2, 3, 4, where *Х*_*К*_ = *m* (KOH)/*m* (C).

The resulting mixture was thoroughly stirred for 1 to 2 h, after which it was dried in the thermostat to constant weight at a temperature of 90°C. The dry material was placed in a furnace and heated in argon atmosphere at 850°C to 920°C with the heating rate of 10°C/min and kept at this temperature for 20 min. After cooling, the resulting material was washed with 5% aqueous HCl and distilled water to neutral pH and again dried at 90°C to constant weight. The samples are numbered according to the ratio of KOH and C (C31, C32, C33, and C34). For example, C32 is a material carbonized at 300°C and mixed with potassium hydroxide at a ratio of 1:2.

The characteristics of porous structure (surface area and total pore volume) of NCM were determined by analyzing the adsorption/desorption isotherms of nitrogen at its boiling point (77 Κ), obtained using Quantachrome Autosorb Nova2200e (Quantachrome Instruments, Boynton Beach, FL, USA). Before taking the measurements, the samples were degassed at 180°C for 18 h. The specific surface area (*S*_BET_, m^2^/g) was determined by multipoint BET method in limited range of relative pressure *P*/*P*_0_ = 0.050 … 0.035. The total pore volume (*V*_total_, cm^3^/g) was calculated by the number of adsorbed nitrogen at *P*/*P*_0_ ~ 1.0. The volume of micropores (*V*_micro_, cm^3^/g), the values of specific surface of micro (*S*_micro_, m^2^/g), and mesopores (*S*_mezo_, m^2^/g) were found using the t-method [6].

The structural study was conducted with scanning electron microscope JSM-6700 F (JEOL, Peabody, MA, USA) with energy system JED-2300. For scanning electron microscopy and X-ray microanalysis, the thin layer of the sample was put into the epoxy resin piece. The sample size does not exceed *D* = 25 mm at a height of 10 mm.

The electrodes of EC were prepared of a lamellar form of a mixture < NCM>:<CA > = <75>:<25>, where CA is a conductive additive (KS-15 graphite (Lonza Group, Basel, Switzerland)). These symmetrical electrodes were infiltrated by the electrolyte and were separated by a separator and sealed in two-electrode cell of the size ‘2525’. As the electrolyte 30% KOH solution was used.

The investigation of electrochemical properties of the EC was conducted with galvanostatic and potentiodynamic cycling and electrochemical impedance spectroscopy (EIS) in the frequency range of 10^−2^ to 10^5^ Hz. The measurements were carried out on a set Autolab PGSTAT12 of ECO CHEMIE Company (Utrecht, Netherlands), equipped with software GPES and FRA-2.

Galvanostatic measurements were conducted in voltage range of 0 to 1 V and charge/discharge current changed within 10 to 100 mA. The specific capacity was calculated by the formula: *C* = 2*I* ⋅ *t*_*d*_/[(*U*_*m*_ − *ΔU*) ⋅ *m*], where *I* is a charge/discharge current, *t*d is the discharge time, *U*m is the maximum voltage, *ΔU* is the voltage drop at the closure of the discharge circle, and *m* is weight of the NCM. The IR was determined by the potential jump after ten charge/discharge cycles: *ΔU* = *IR*.

The values of specific capacity according to potentiodynamic measurements were calculated as *C* = 2*I*/*s* ⋅ *m*, where *I* is a current in anodic or cathodic branches of voltammograms, *s* is a scan rate, and *m* is weight of the NCM.

According to the EIS, the specific capacity of the electrochemical capacitor reduced to weight unit of active electrode was calculated by the formula: *C* = 1/(2*mf* ⋅ Im*Z*), where *f* is a frequency, *Im Z* is imaginary part of the impedance, and *m* is weight of the NCM. EIS data were modeled with standard electrical equivalent circuit (EEC) using the software ZView-2.

## Results and discussion

**Figure 1:**
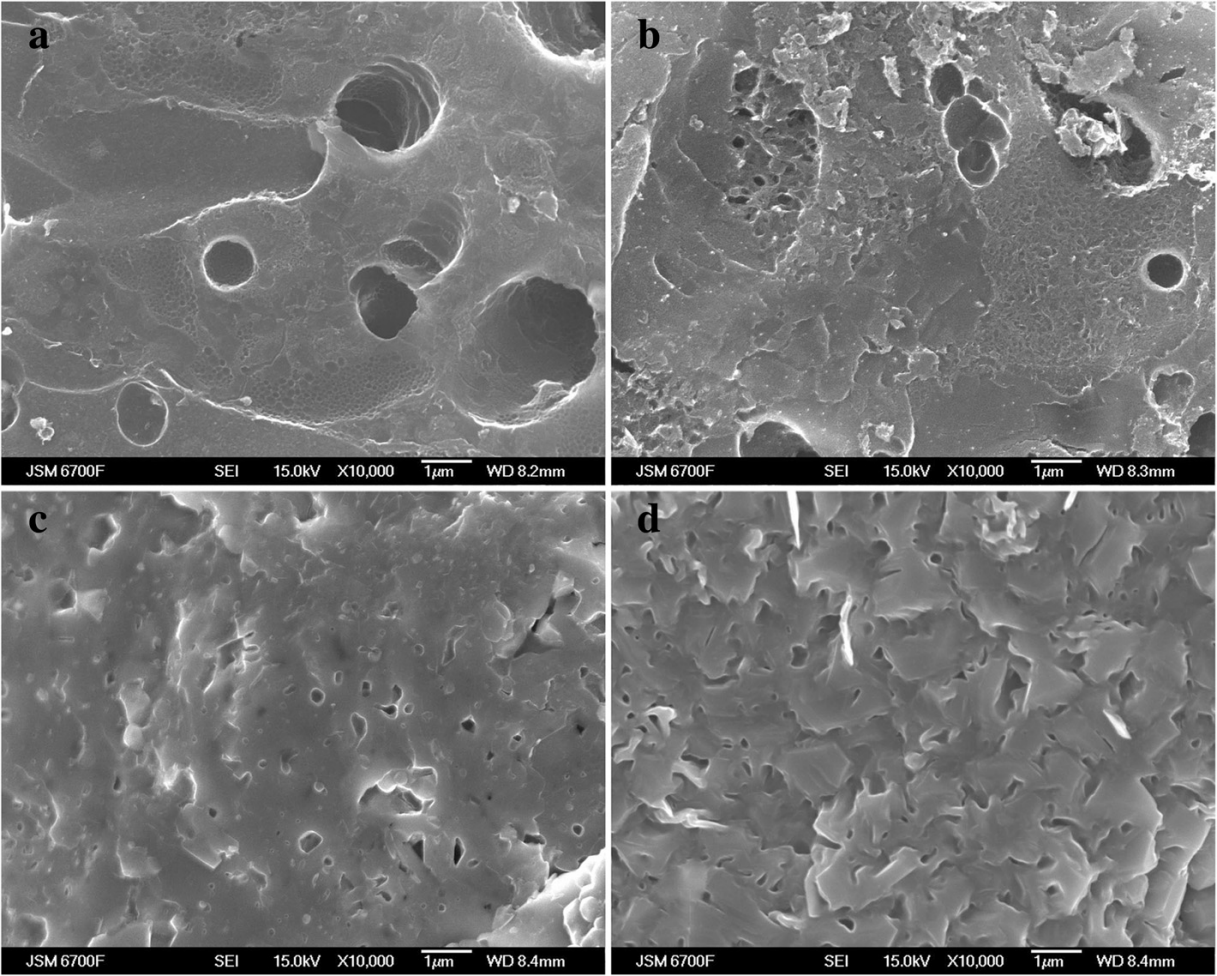
**The microstructure of sample surfaces of С31 (а), С32 (b), С33 (c), and С34 (d).**

The main method for NCM receiving is the carbonation of a feedstock at different temperatures, followed by chemical activation. The output of the final product and its structure (Figure 1) depends on the ratio of the initial component (KOH:C), activation temperature, and time.

When considering the sample surface C31 (Figure 1a), the presence of the surface microcracks and round or oval transport pores with size about 0.1 to 2 μm is visible, resulting from the reaction of KOH with carbon and organic residues. These remain in almost the whole natural structure of the fruit seed. For this sample, the typical is the chemical homogeneity of the surface. With the increasing KOH concentration, on the surface of the sample C32 (Figure 1b), there is formation of new pores and coal fragments, which is the beginning of the pore walls' destruction and the surface corrosion of the carbon particles. For samples C33 and C34, the surface melting is observed, leading to the closure of the external pores. The surface becomes smoother.

**Figure 2:**
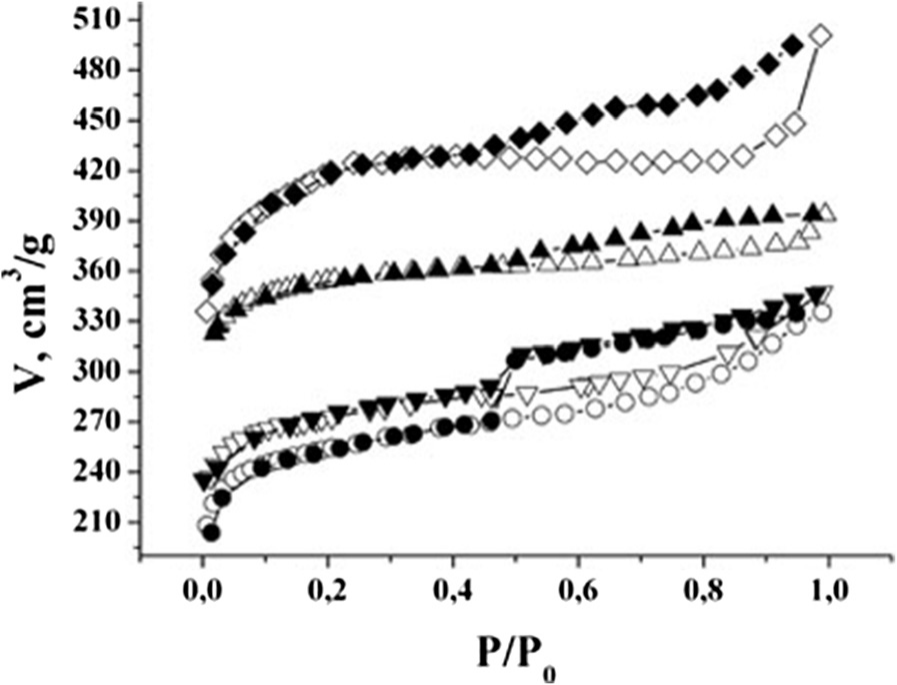
**Adsorption/desorption isotherms of nitrogen.** С31 —●—, С32 —▼—, С33 —▲—, С34 —♦— (hollow symbols, аdsorption; filled symbols, desorption).

The typical adsorption/desorption isotherms of nitrogen at 77 K for NCM are shown in Figure 2. For all sample isotherms, the hysteresis loop of H3 type is observed according to IUPAC classification [6]. This type of sorption isotherms associates with processes in micropores and capillary condensation in meso- and macropores of organic origin materials and adsorption on the external surface of the particles [7].

The analysis of sorption isotherms made it possible to determine the parameters of the porous structure of the samples (Table 1). As the table shows, sample C34 has the greatest specific surface area; it has the most developed microporous structure, which is 92% of the total surface area.

**Table 1:** **Structural and adsorption characteristics of NCM**

**Sample**	***S***_BET_ **(m** ^**2**^ **/g)**	***S***_micro_ **, (m** ^**2**^ **/g)**	***V***_micro_ **, (cm** ^**3**^ **/g)**	***S***_meso_ **, (m** ^**2**^ **/g)**	***V***_total_ **, (cm** ^**3**^ **/g)**
С31	950	817	0.33	133	0.52
С32	1,030	915	0.369	114	0.537
С33	1,340	1,266	0.51	74	0.58
С34	1,553	1,426	0.546	127	0.774

By increasing the ratio of KOH/C, the number of micropores increases, but the percentage of mesopores decreases, which serve as transport channels for electrolyte penetration into the micropores.

The accumulation of electric charge in EC based on NCM in aqueous electrolyte is mainly due to EDL charge/discharge formed at the interface electrode║electrolyte. However, on the surface of the carbon material, Faraday processes of electricity accumulation occur through redox reactions [8]. As a result, there are free electrons, which are also involved in the formation of EDL.

**Figure 3:**
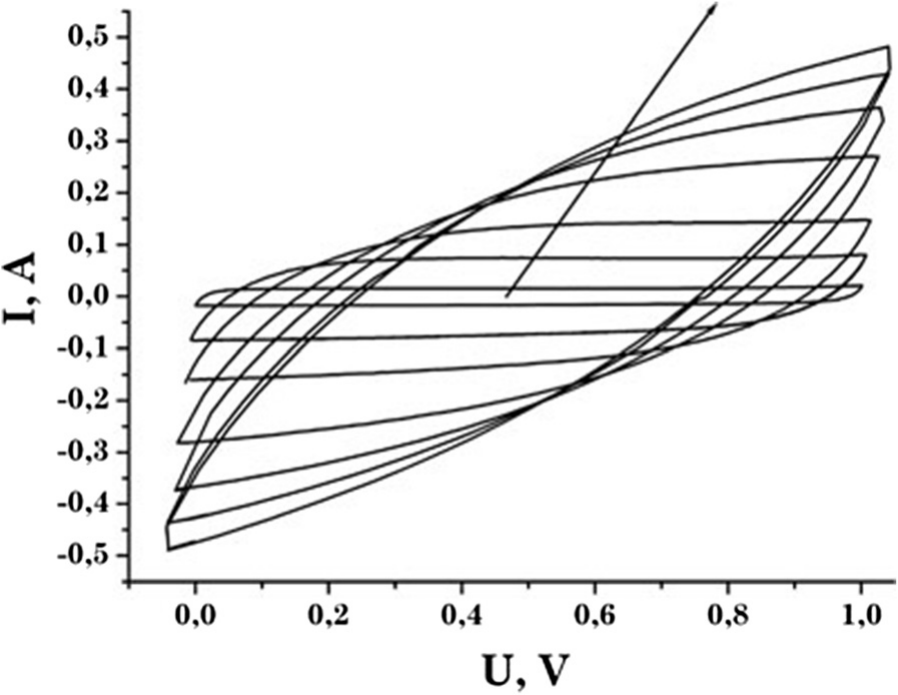
**Cyclic voltammograms of the sample C31.** Scan rate was 1, 5, 10, 20, 30, 40, 50 mV/s. The arrow indicates the increasing direction of the scan rate.

In order to detect the possible occurrence of chemical reactions that contribute to the overall capacity of the condenser systems, potentiodynamic research was conducted in the potential range of 0 to 1 V. Figure 3 shows the cyclic voltammograms of EC formed from material C31. The cyclic voltammograms of other samples are similar to C31. All specimens exhibit ideal polarization at low scanning rate (*s* = 1 to 10 mV/s).

With increasing scan rate, an insignificant distortion of voltammograms and its deviation from the rectangular shape can be associated with the increase of the EC IR. This is the initial stage of the ‘fasting electrolyte’ effect when the electrolyte ions are removed by their adsorption on the EDL edges [9]. It should also be noted that none of the curves, both positive and negative branches of the voltammograms, has visible peaks, which are the characteristic especially for battery cells [10]. Therefore, we can mention the chemical and electrochemical stability of the system electrode/electrolyte. For all samples, the current increases with the increasing scan rate. For the sample C31 at scan rate of 20 mV/s, the current value is 1.5 times larger than the corresponding values of currents for samples C33 and C34.

**Figure 4:**
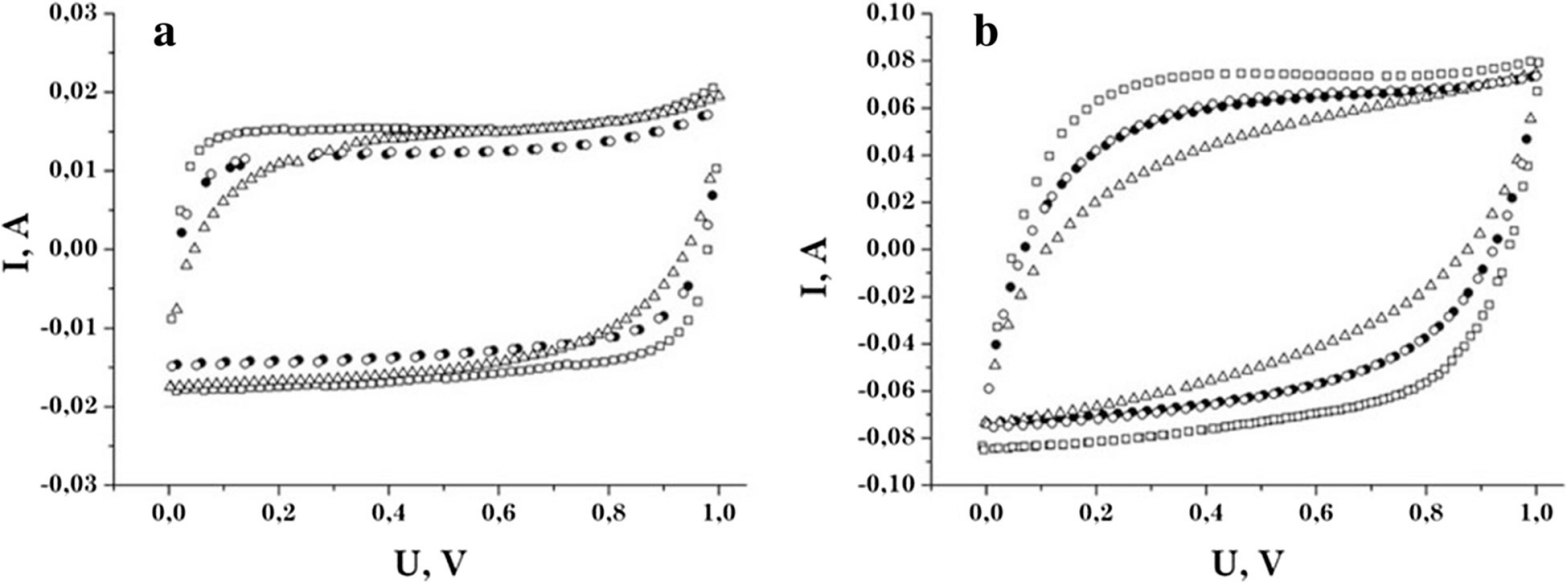
**Cyclic voltammograms of the NCM taken at different scan rates. (а)** *s* = 1 mV/s. **(b)** *s* = 5 mV/s. C31 —□—, C32 —●—, C33 —Δ—, C34 —○—.

Figure 4 shows the cyclic voltammograms for the NCM in 30% aqueous KOH at the linear scanning electrode potential of 1 and 5 mV/s. When scan rate is *s* = 1 mV/s, the curves have a symmetrical, almost rectangular shape with no obvious redox peaks, indicating the dominance of the electrostatic electric charge accumulation processes at the interface electrode/electrolyte [11]. A small peak at the potentials of 0.85 … 1 V is due to the release of oxygen dissolved in the electrolyte and adsorbed by the surface of the active material [12]. Comparing the cyclic voltammograms at the scan rate of 5 mV/s, it can be noted that all the samples in the positive potential range have a slight peak. Given that in this region the capacity of the material is provided mostly by negative electrolyte ions (OH^−^ groups), we can conclude that there is a possible intercalation process of these groups into the pores of the electrode material. Using these cyclic voltammograms, we calculated the values of the specific capacity of the samples at the scan rate of 1 mV/s (Table 2).

**Table 2:** **Specific capacity of the investigated NCM, F/g**

	**Methods of the study**		
	**Chrono-potentiometry**	**Voltammetry**	**Impedance spectroscopy**
C31	178	168	169
C32	156	152	154
C33	162	160	163
C34	165	164	163

**Figure 5:**
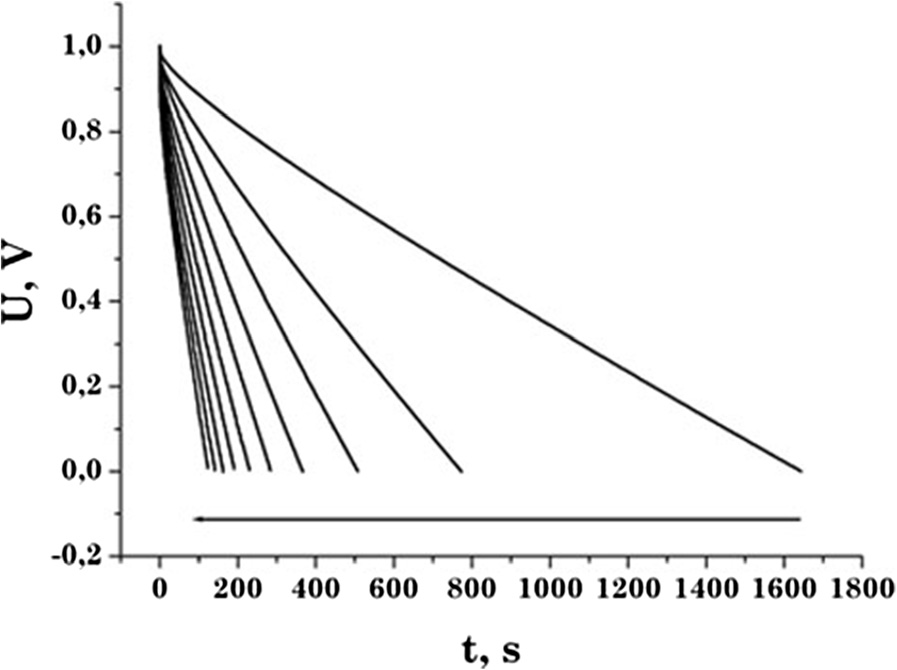
**Discharge curves of the EC based on the carbon material C31.** The arrow indicates the direction of growth of the discharge current from 10 to 100 mA in step of 10 mA.

According to the galvanostatic studies, the sample C31 has the largest capacity (Table 2). The discharge curves show a linear voltage dependence of the discharge current, which is a characteristic of the capacitive behavior of the supercapacitors formed on the studied material (Figure 5).

According to the results of galvanostatic study, the values of specific capacity at the discharge current of 15 mA are obtained (Table 2). These values of supercapacitors obtained by this method coincide with the results that were obtained by voltammetry within an acceptable error.

**Figure 6:**
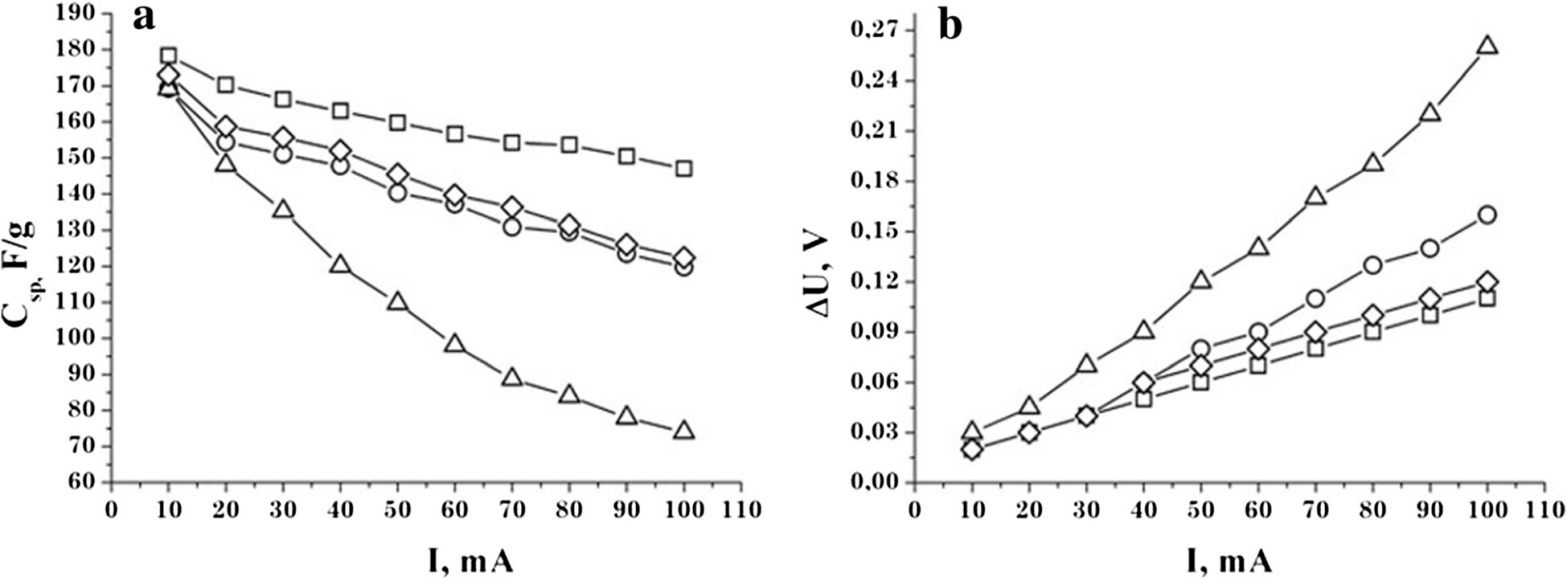
**Dependence of specific capacity NCM (a) and IR drop (b) EC on discharge current.** C31 —□—, C32 — Δ —, C33 —○—, C34 —◊—.

The reduction of the specific capacity of the studied materials with the current density increase (Figure 6a) is due to the fact that not all micropores are involved in the charge/discharge processes of the material surface. The latter fact leads to a gradual increase in the ohmic resistance and, consequently, in the voltage drop of the EC at the discharge (Figure 6b).

For all samples, there is a monotonous capacity decrease with the increasing discharge current. For sample C32 with the increasing current from 10 to 100 mA, the capacity decreased by 53%. The sample C31 has the smallest capacity decline because the specific capacity at the discharge current 10 mA was 178 F/g and the change in capacity with the increasing current up to 100 mA does not exceed 18%. Figure 6b also shows that for samples C31, C33, and C34, the voltage drop at maximum discharge current 100 mA does not exceed 14%. A characteristic feature for this group of samples is the fact that at discharge current of 10 to 30 mA, the IR drop is almost the same, indicating the same number of free charge carriers that participate in the EDL formation. The largest IR drop (24%) was observed for EC based on sample C32.

**Figure 7:**
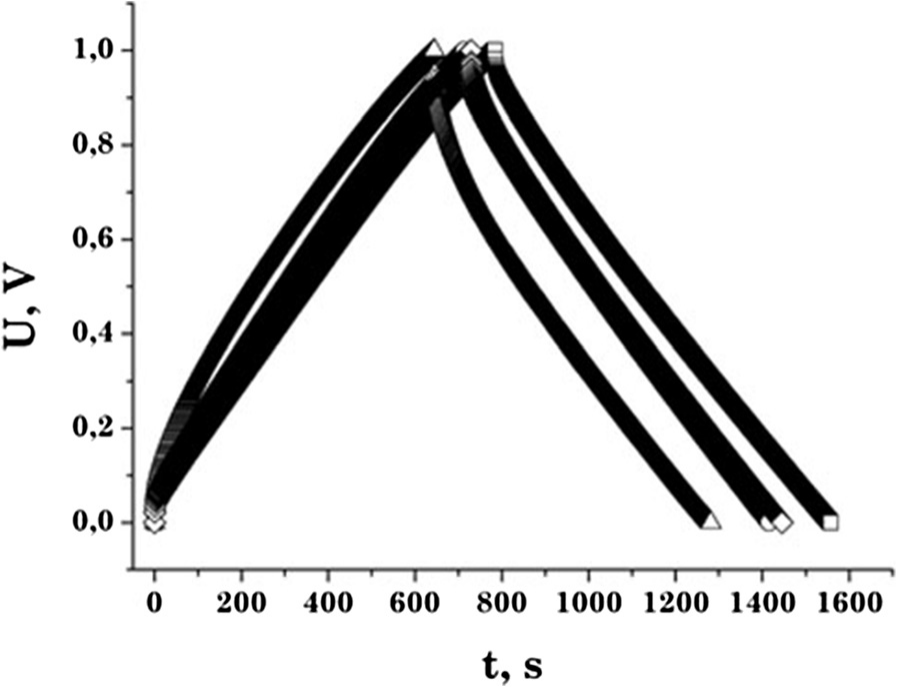
**The charge-discharge curves of the EC based on the NCM.** C31 —□—, C32 —Δ —, C33 —○—, C34 —◊—. (Discharge current is 20 mA).

One of the important parameters of EC is Coulomb efficiency, which indicates the ratio of the discharge capacity to charge capacity during cycling. In order to determine this parameter, we took the charge/discharge curves of EC after 1,000 cycles (Figure 7). The Coulomb efficiency after several cycles stabilized at the level of 97% to 99% and practically did not change during the study. It shows that the studied material provides stability behavior and durability of capacitors. The capacity change during cycling is less than 2%.

To study the electrochemical processes at the interface between the NCM and the electrolyte, the EIS method was used. It allowed establishing the correlation between the internal structure of the carbon electrode material and its behavior in aqueous electrolyte solution.

This method is most appropriate for solving the abovementioned problems, since it allows conducting research in a wide frequency range (10^−3^ to 10^6^ Hz) [13].

**Figure 8:**
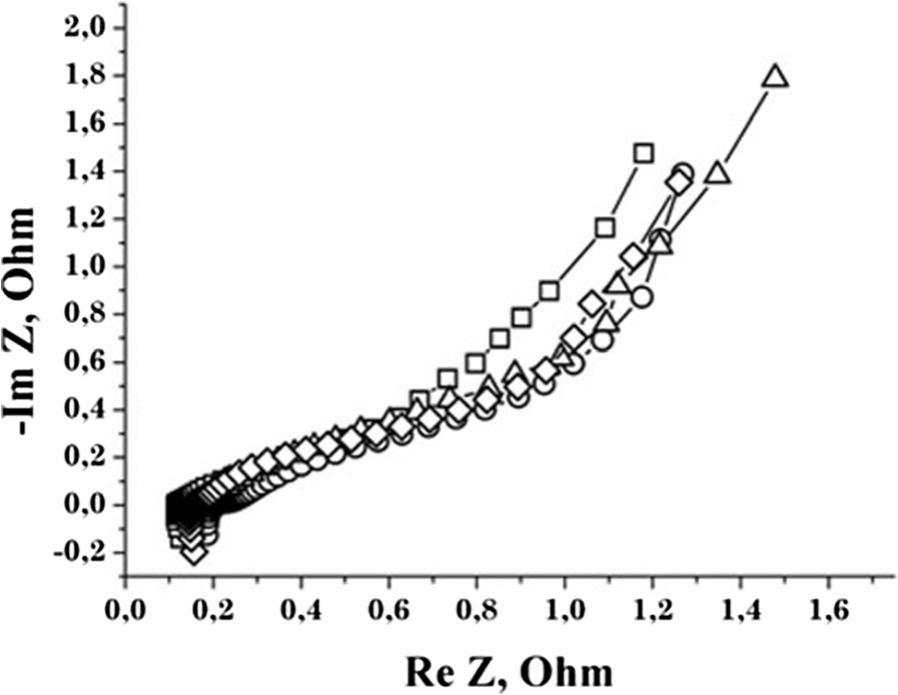
**The Nyquist diagrams for capacitor systems based on NCM.** C31 —□—, C32 — Δ—, C33 —○—, C34 —◊—.

The structural modeling of a predictable processes based on the experimental data obtained by EIS is a systematic approach in which the object is seen as EEC, which includes elements that characterize the boundary phase of electrode/electrolyte. EEC is a simplified model of real processes in the studied system, which creates resistance to electric current. The main criterion for the choice of the equivalent circuit is a complete physical design of all its structural elements providing the optimal approximation of experimental impedance hodograph − Im*Z* = *f*(Re*Z*) (Figure 8).

**Figure 9:**
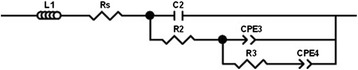
**The EEC obtained for the system NCM-aqueous electrolyte.**

Therefore, to approximate the experimental curves that describe the process of electrode polarization at all stages of electrochemical implementation, we used EEC, shown in Figure 9. In this scheme, the element *L*_1_ reflects the inductive behavior of the electrochemical system, which is due to the metal of the case. Resistance *R*_s_ includes the resistance of the electrolyte, contacts, and supply cables. The following circuit elements are responsible for the diffusion processes in the pores of the carbon material and the accumulation of electric charges at the interface electrolyte-carbon. In particular, the link *C*_2_║*R*_2_ is responsible for the processes in the transport pores, which include macro-and mesopores in the NCM, the link *CPE*_3_║*R*_3_-*CPE*_4_ in the micropores. The use of this EEC has made it possible to approach quite well the experimental spectrum to calculate one and to obtain the parameters that the scheme includes (Table 3). The Kramers-Kronig coefficient does not exceed 5 × 10^−4^; the difference between experimental and model curves does not exceed 5%. As follows from the data in Table 3, the values of *L*_1_ and *R*_s_ are virtually unchanged. It is due to the electrochemical cell configuration immutability and constancy of the concentration of the electrolyte solution. The increase in capacity of *C*_2_ and the corresponding decrease in the resistance *R*_2_ are, most likely, due to the increase in the proportion of macro-and mesopores in NCM in the total number of pores through the thermochemical processing. To simulate the impedance hodograph, we used a constant phase element (CPE), the impedance of which is defined by equality ${Z}_{CPE}=CP{E}_T{\left(j\omega \right)}^{-CP{E}_P}$ [13]. This CPEР takes into account the phase deviation and, consequently, the type of modeled process. When the value of CPEР is close to 1, this element describes the capacitive behavior of the system, CPEP ~ 0.5 of the diffusion. The introduction of this element is caused by the fractal structure of the NCM [14] and the inhomogeneous distribution of charge carriers at the interface electrode-electrolyte. In general terms, it is considered as a result of diffusion to the unequally available surface, which formally attributed to fractional dimension (fractal surface) or as a direct charge accumulation on the surface. In this case we got a distorted capacity. This was the form that the CPE element is represented in most publications. Quite often, this property is identified as the surface roughness.

**Table 3:** **Options' EEC modeling of the electrochemical processes in EC**

**Sample**	***L***_1_ **(μH)**	***R***_s_ **(Ω)**	***C***_2_ **(mF)**	***R***_2_ **(mΩ)**	**CPE**_3_ **-** ***T*** **(Ω)**	**CPE**_3_ **-** ***P***	**R**_3_ **(Ω)**	**CPE**_4_ **-** ***T*** **(F)**	**CPE**_4_ **-** ***P***
C31	0.24	0.17	0.008	46	1.44	0.55	1.001	5.732	0.89
С32	0.20	0.19	0.291	40	1.21	0.53	1.894	5.312	0.99
С33	0.19	0.15	0.680	18	1.02	0.58	1.817	3.488	0.89
С34	0.22	0.15	11.18	13	0.87	0.64	1.056	5.14	0.81

Based on this data and simulation, we see that the СРЕ_3_ element is responsible for the diffusion restrictions on the transport of electrolyte ions into the micropores (the value of the CPE_3_-*P* is close to 0.5), and its behavior is similar to change of the parameter *R*_2_. In our opinion, it is also caused by an increase in the proportion of micropores of the NCM, consequently the deviation of the element behavior from the ideal (for the sample C34, the value of the CPEP is equal to 0.64). The value of the resistance *R*_3_ remains almost unchanged, indicating a negligible effect of the thermochemical processing of the NCM on its electronic configuration and electrical properties. The option CPE_4_ changes more significantly, which according to Table 2 can be attributed to the steady capacitive phase (the value of the CPE_4_-*P* is close to 1), which reflects the capacity of the EDL at the interface electrolyte-NCM in micropores.

## Conclusions

Based on the analysis of the results, it has revealed that one of the best ways to get a carbon electrode material is the carbonization of the feedstock of the plant origin in a closed furnace at 250°C to 350°C and the following chemical activation with potassium hydroxide in the different ratios of KOH/C in the temperature range 850°C to 920°C.

According to the electrochemical studies, we found that an increase in the percentage of KOH during chemical activation leads to a decrease in specific capacity of the NCM by 8% to 12% and an increase in the IR of EC by 10%.

Using synthesized activated carbon as electrode material of EC with aqueous electrolyte (KOH) provides a maximum value of the specific capacity of 160 to 180 F/g in the range of operating currents (10 to 100 mA) at a maximum charging voltage of 1 V.

For supercapacitors based on NCM, we chose the EEC diagram, which is describing the processes occurring at the interface electrode/electrolyte and is consistent with the results of EIS.

## Abbreviations

EC: electrochemical capacitors
NCM: nanoporous carbon material
EDL: electrical double layer
EIS: electrochemical impedance spectroscopy
EEC: electrical equivalent circuit
